# NDUFB11 and NDUFS3 play a role in atherosclerosis and chronic stress

**DOI:** 10.18632/aging.204947

**Published:** 2023-08-28

**Authors:** Yin Yang, Jing-Hui Li, Bo-Chen Yao, Qing-Liang Chen, Nan Jiang, Lian-Qun Wang, Zhi-Gang Guo

**Affiliations:** 1 Clinical School of Thoracic, Tianjin Medical University, Tianjin Chest Hospital, Jinnan, Tianjin 300222, P.R. China

**Keywords:** NDUFB11, NDUFS3, atherosclerosis, chronic stress, bioinformatics

## Abstract

Objective: Atherosclerosis is characterized by the formation of fibrofatty plaques in the intima of arteries, resulting in thickening of the vessel wall and narrowing of the lumen. Chronic stress refers to individuals in a state of long-term chronic stress. However, the relationship between NDUFB11 and NDUFS3 and atherosclerosis and chronic stress is unclear.

Method: The atherosclerosis with chronic stress group data file was used. DEGs were screened and WGCNA was performed. Construction and analysis of PPI Network. Functional enrichment analysis, GSEA, gene expression heatmap, immune infiltration analysis and mRNA analysis were performed. CTD was used to find diseases most related to core genes. WB was performed. TargetScan was used to screen miRNAs of DEGs.

Results: 1708 DEGs were identified. According to GO analysis, they were mainly enriched in catabolic processes, organic acid metabolism processes, carboxylic acid metabolism processes. KEGG analysis showed that they were mainly enriched in metabolic pathways, fatty acid metabolism, pentose phosphate pathway, glycolysis / gluconeogenesis, fructose and mannose metabolism. Gene expression heatmap showed that the core genes (NDUFB11, NDUFS3) were lowly expressed in samples of those with atherosclerosis accompanied by chronic stress and highly expressed in the normal samples. NDUFB11 and NDUFS3 were associated with necrosis, hyperplasia, inflammation, renal disease, weight loss, memory impairment, and cognitive impairment. WB showed that the expression level of NDUFS3 in atherosclerosis and chronic stress was lower than that in control group.

Conclusions: NDUFB11 and NDUFS3 are underexpressed in atherosclerosis and chronic stress; the lower NDUFB11 and NDUFS3 levels, the worse the prognosis.

## INTRODUCTION

Atherosclerosis is a chronic disease caused by the deposition of lipids, cholesterol, and other substances on the inner wall of blood vessels, as well as endothelial cell damage due to inflammatory reactions [1]. Atherosclerosis usually develops over age of 40, and men are at higher risk of developing atherosclerosis [2]. Atherosclerosis is mainly characterized by lipid deposition and inflammatory reactions on the intimal layer of blood vessels, which progressively lead to the thickening and loss of elasticity of the intimal layer and eventually the formation of arterial plaques. Atherosclerosis is a chronic progressive disease. The clinical manifestations of atherosclerosis may be asymptomatic in the early stages, but as the disease progresses, cardiovascular, cerebrovascular, peripheral vascular, renal lesions may develop [3, 4]. Chronic stress refers to a state in which an individual is stimulated by persistent psychological or physical stress over a period of time [5]. Studies have shown that young people under 20 years of age, those with high intensity, long hours and high professional responsibilities, and those with low income and educational levels are more susceptible to chronic stress [6]. Chronic stress is a chronically accumulated stress that may affect multiple aspects of physical and mental health and may affect different individuals [7]. The pathological features of chronic stress are increased or decreased secretion of neuroendocrine hormones causing changes in the cardiovascular, immune, and metabolic systems, decreased immune function or dysregulated immune responses, increased oxidative stress damaging cells and tissues, affecting energy metabolism, and increased inflammatory responses [8]. The causes of atherosclerosis and chronic stress are not clear, and genetic factors, chromosomal abnormalities, and gene fusions may contribute to this disease. Therefore, it is particularly important to deeply investigate molecular mechanisms of atherosclerosis and chronic stress.

Bioinformatics is an interdisciplinary field that involves computer science, mathematics, biology, and statistics [9]. The development of bioinformatics technology has greatly assisted biological research, accelerating the interpretation and understanding of biomolecules such as genomes, proteins, and metabolomes. Bioinformatics technology includes sequence analysis, structure analysis, functional prediction, systems biology, genomics, and proteomics. The advantages of bioinformatics technology are mainly reflected in its efficiency, accuracy, visualization, and reproducibility [10, 11].

However, the relationship between NDUFB11 and NDUFS3 and atherosclerosis and chronic stress is still unclear. Therefore, we wanted to use bioinformatics to mine core genes between atherosclerosis and chronic stress and normal tissues, perform enrichment analysis, pathway analysis. Validation of significant role of NDUFB11 and NDUFS3 in atherosclerosis and chronic stress using public datasets. And the basal cell experiment was applied to verify it.

## RESULTS

### Differential gene analysis

**Figure 1:**
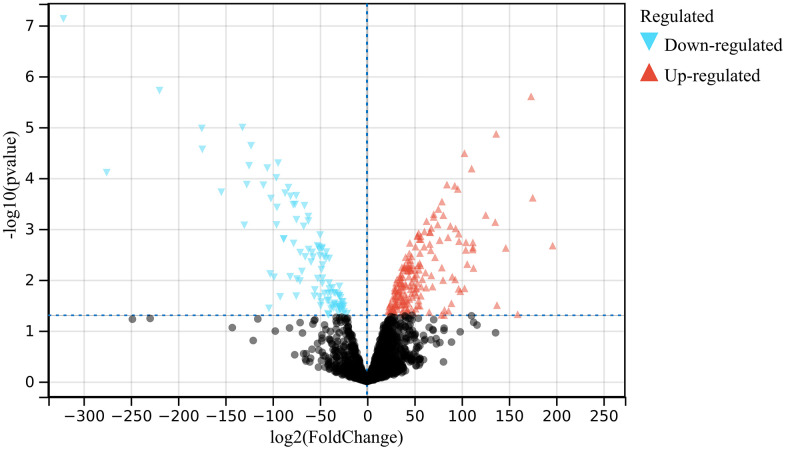
**Analysis of differentially expressed genes.** 1708 DEGs were identified.

In the present study, following the set cut-off value, 1708 DEGs were identified based on identification of differentially expressed genes in the atherosclerosis accompanying chronic stress matrix (Figure 1).

### Functional enrichment analysis

### DEGs

**Figure 2:**
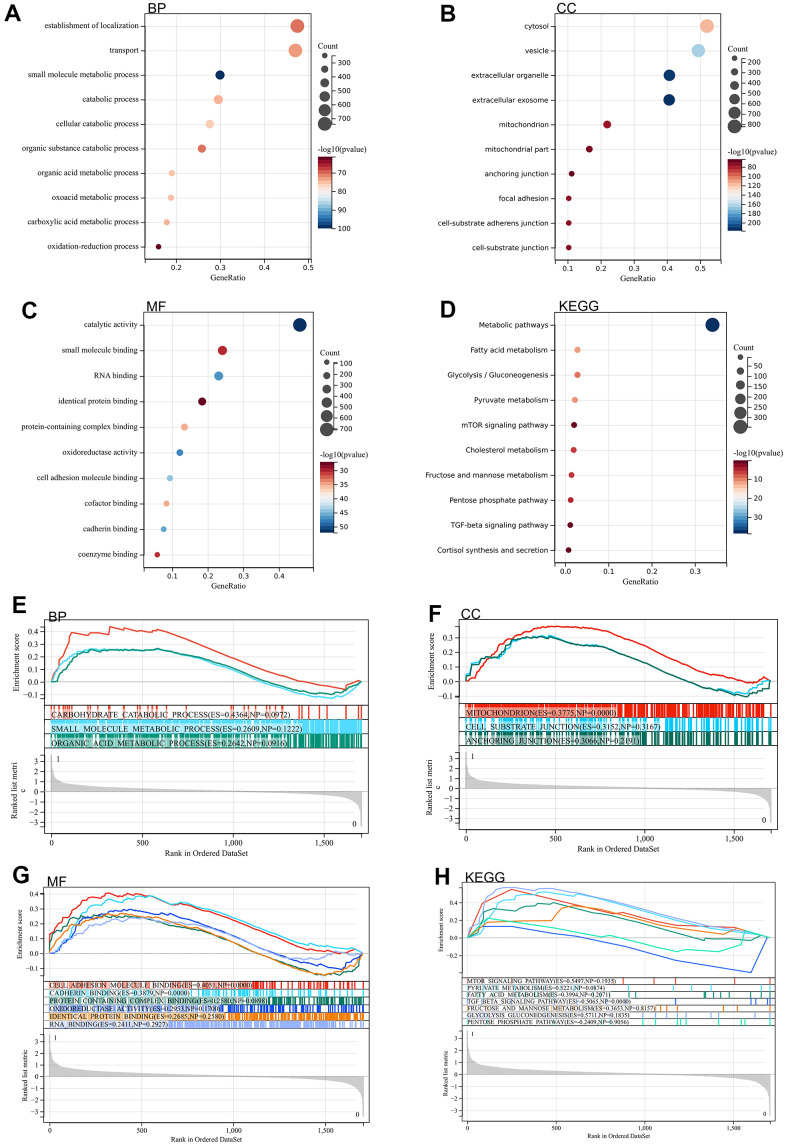
**Functional enrichment analysis.** (**A**–**D**) DEGs (**E**–**H**) GSEA.

We analyzed DEGs by GO and KEGG. According to GO analysis, they were mainly enriched in catabolic processes, organic acid metabolism processes, carboxylic acid metabolism processes. Through KEGG analysis, they were mainly enriched in metabolic pathways, fatty acid metabolism, pentose phosphate pathway, glycolysis / gluconeogenesis, fructose and mannose metabolism (Figure 2A–2D).

### GSEA

A genome-wide GSEA was performed to find possible enrichment terms among the non-differentially expressed genes and to validate the differentially expressed genes. The intersection of the enriched terms with the GO KEGG enriched terms of the differentially expressed genes is shown in, and was mainly enriched in the pentose phosphate pathway, fatty acid metabolism, glycolysis / gluconeogenesis (Figure 2E–2H).

### Metascape enrichment analysis

**Figure 3:**
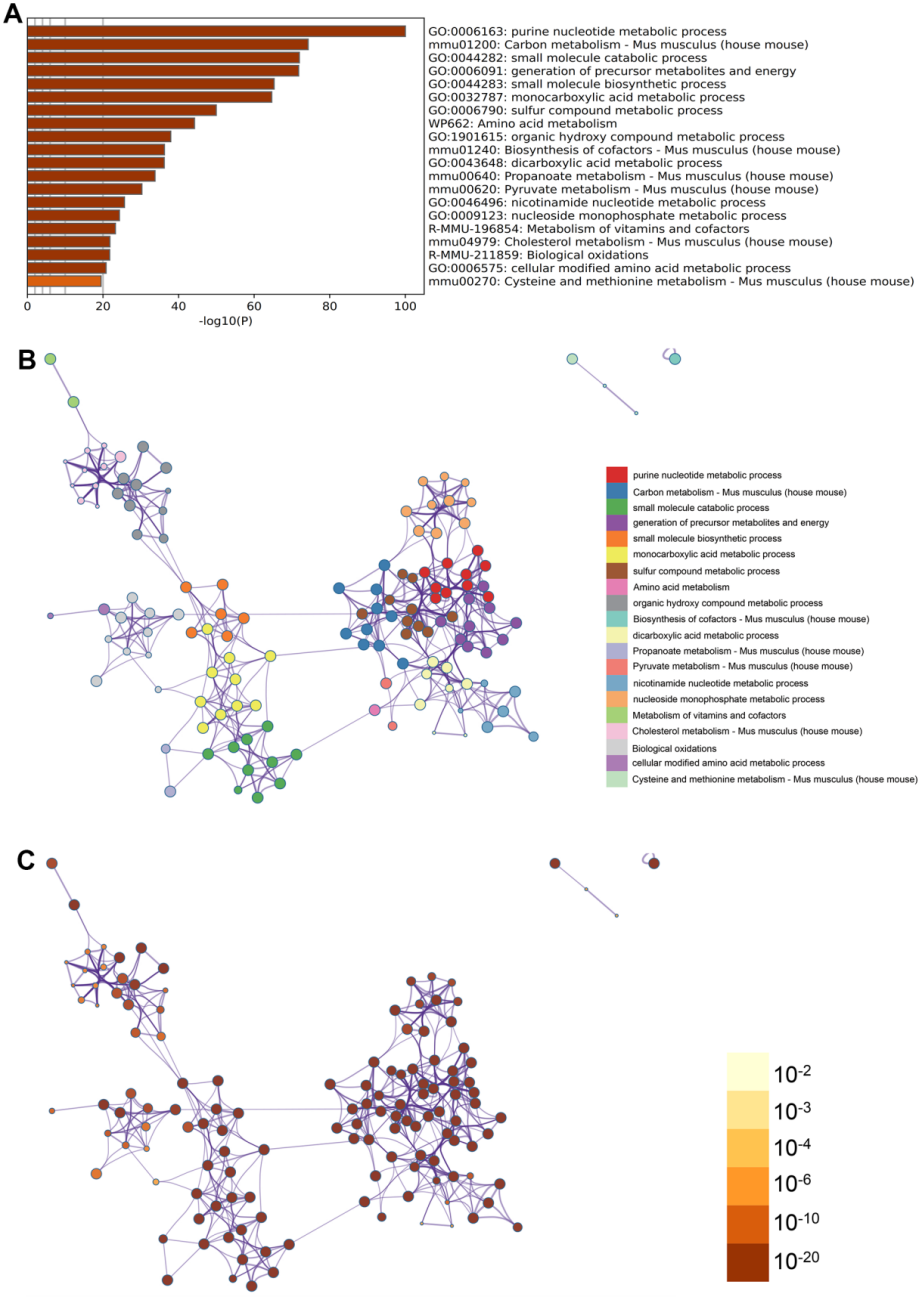
**Metascape enrichment analysis.** (**A**) Purine nucleotide metabolic process, monocarboxylate metabolic process, amino acid metabolism were found in GO enrichment items in the enrichment items of metascape (**B**) output the enrichment network colored by enrichment terms (**C**) output the enrichment network colored by p-value.

**Figure 4:**
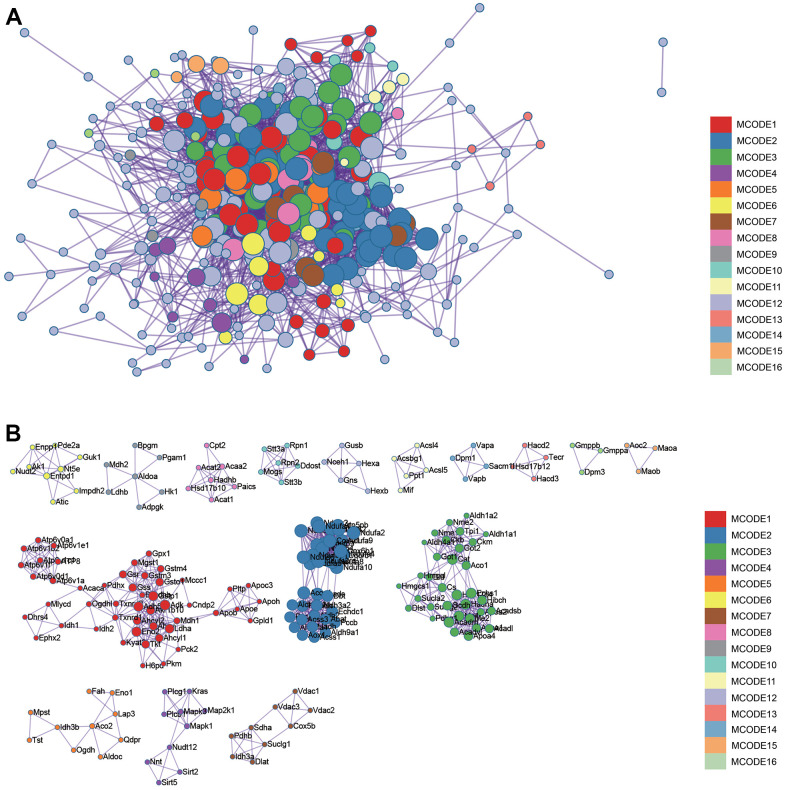
**Metascape enrichment analysis.** Visualize the association and confidence representing each enrichment item. (**A**) Protein-protein interact by the Metascape. (**B**) The significant modules.

Purine nucleotide metabolic process, monocarboxylate metabolic process, amino acid metabolism were found in GO enrichment items in the enrichment items of metascape (Figure 3A), meanwhile we also exported the enrichment network colored by enrichment term and p-value (Figures 3B, 3C, 4) to visualize the association and confidence representing each enrichment item.

### WGCNA

**Figure 5:**
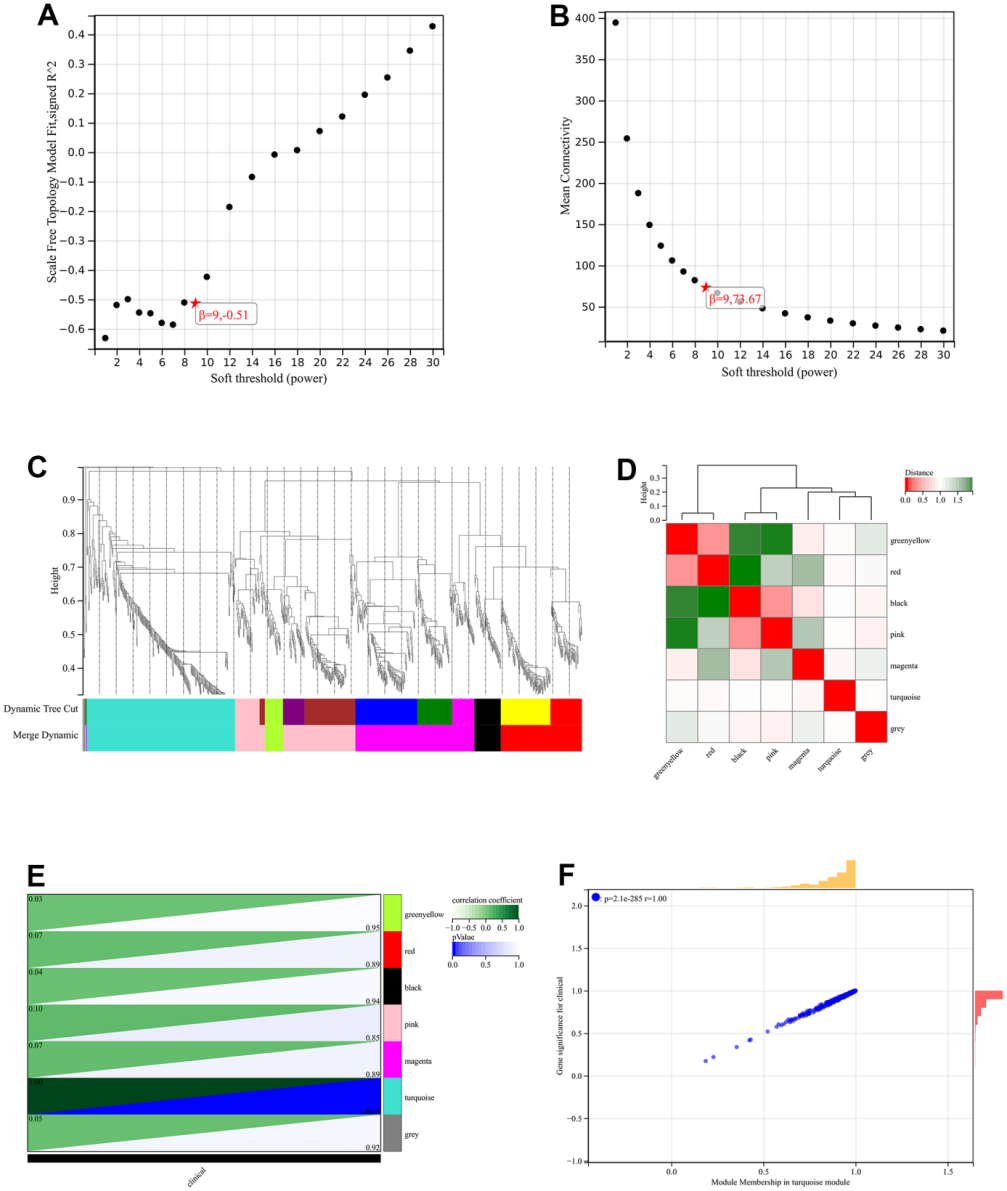
**WGCNA.** (**A**) β=6, 0.70 (**B**) β=6, 163.48 (**C**) A hierarchical clustering tree of all genes was constructed, and 3 significant modules were generated (**D**) Interactions between these modules were then analyzed (**E**) Generated a module to phenotype correlation Heatmap (**F**) GS to MM correlation scatter plot of the associated hub genes.

The network topology is analyzed and the soft threshold power of WGCNA is set to 9, which was the lowest power for a scale-free topology fit index of 0.9 (Figure 5A, 5B). A hierarchical clustering tree of all genes was constructed, and 3 significant modules were generated (Figure 5C). The interaction between modules was analyzed (Figure 5D). And generated a module to phenotype correlation Heatmap (Figure 5E). GS to MM correlation scatter plot of the associated hub genes (Figure 5F).

### Construction and analysis of protein-protein interaction (PPI) networks

**Figure 6:**
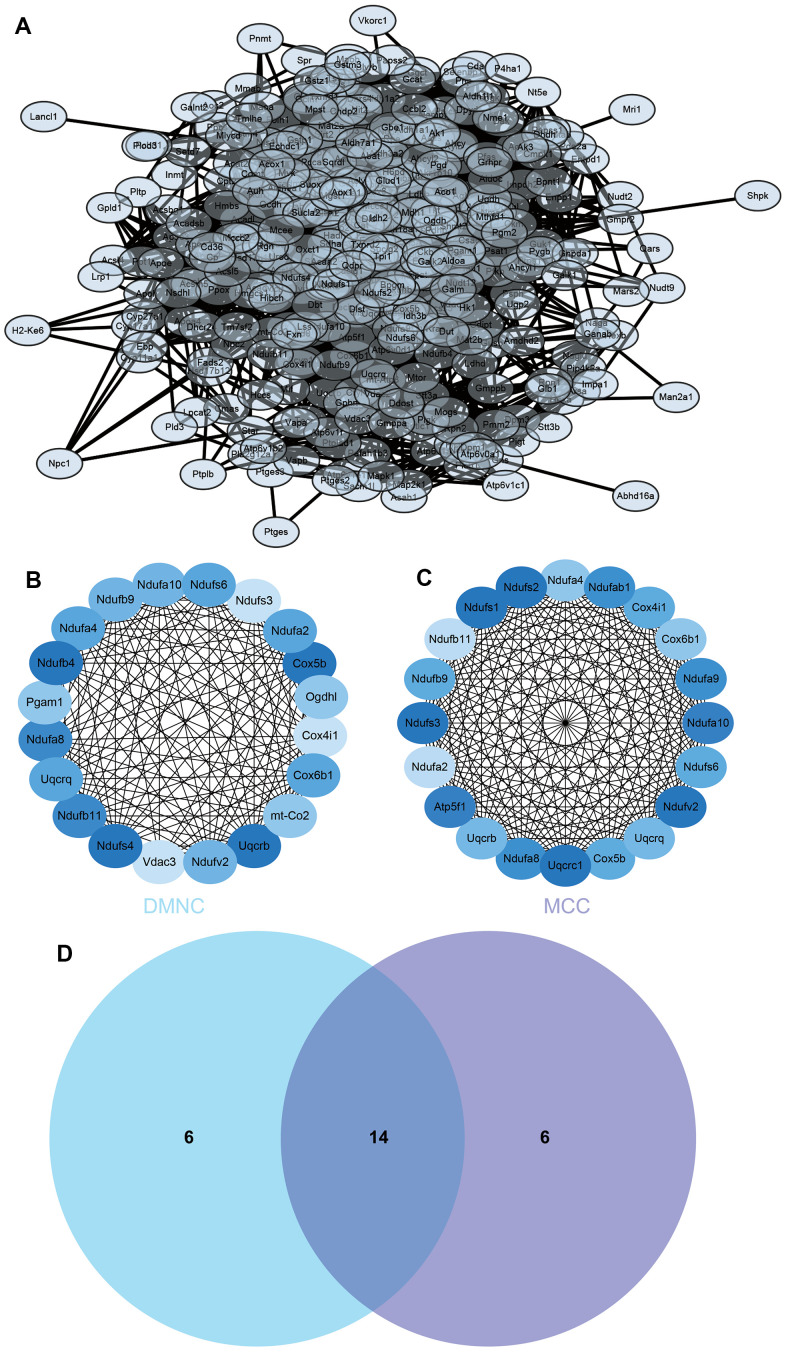
**Construction and analysis of protein-protein interaction (PPI) networks.** (**A**) The PPI network (**B**) DMNC algorithm was adopted to identify the core genes (**C**) MCC algorithm was adopted to identify the core genes (**D**) Venn diagram.

The PPI network of DEGs was constructed from the STRING online database and analyzed by Cytoscape software, we selected the enrichment items in the gokegg analysis related to adrenal gland and input the list of differential genes enriched on these enrichment items into STRING, constructed PPI network (Figure 6A), hub genes were identified using two different algorithms (Figure 6B, 6C), which were merged with the Venn diagram (Figure 6D), Fourteen core genes (UQCRB, COX5B, NDUFB11, NDUFA8, NDUFS6, COX6B1, NDUFA4, UQCRQ, 2NDUFA2, NDUFB9, NDUFV2, NDUFA10, COX4I1, NDUFS3) were obtained.

### Gene expression heatmap

**Figure 7:**
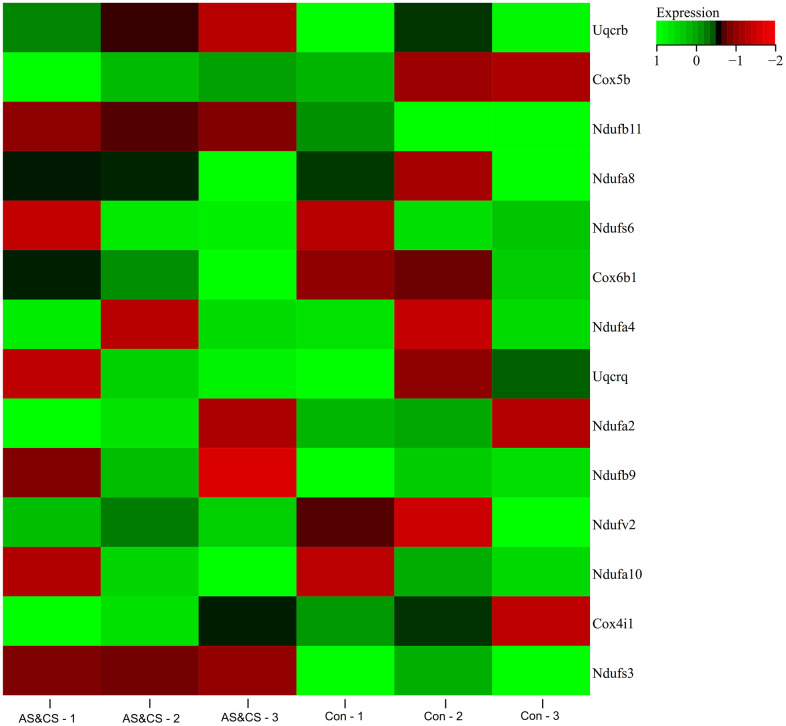
**Gene expression heatmap.** Visualized the expression Heatmap of the core genes in the samples.

A heat map of the expression of core genes in the samples was plotted (Figure 7), and we found that the core genes (NDUFB11, NDUFS3) were lowly expressed in the atherosclerosis accompanying chronic stress samples and highly expressed in the normal samples.

### Immune infiltration analysis

**Figure 8:**
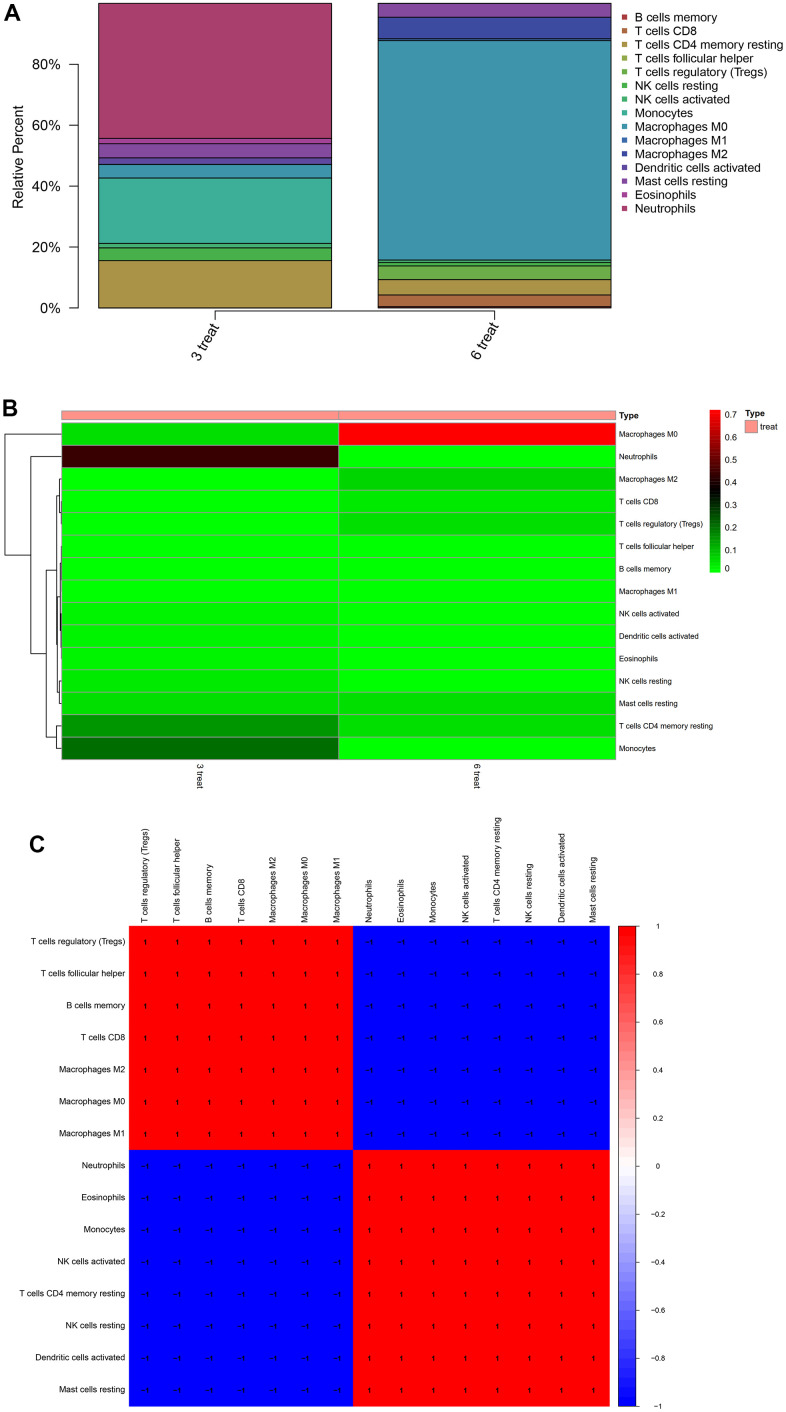
**Immune infiltration analysis.** (**A**) Obtained the proportion results of immune cells from the full gene expression matrix (**B**) the immune cell expression Heatmap in the dataset (**C**) performed the correlation analysis on infiltrated immune cells, resulting in a plot of co expression patterns among immune cell components.

We used the cibersort package to analyze the atherosclerosis accompanying chronic stress matrix file, and at 95% confidence, obtained the proportion results of immune cells from the full gene expression matrix (Figure 8A) and the immune cell expression Heatmap in the dataset (Figure 8B), and also performed the correlation analysis on infiltrated immune cells, resulting in a plot of co expression patterns among immune cell components (Figure 8C).

### The messenger RNA (mRNA) analysis

We obtained autophagy related mRNA differentially expressed genes by extracting autophagy related genes in the matrix of atherosclerosis accompanying chronic stress, intersecting with the differential genes obtained from the screening.

**Figure 9:**
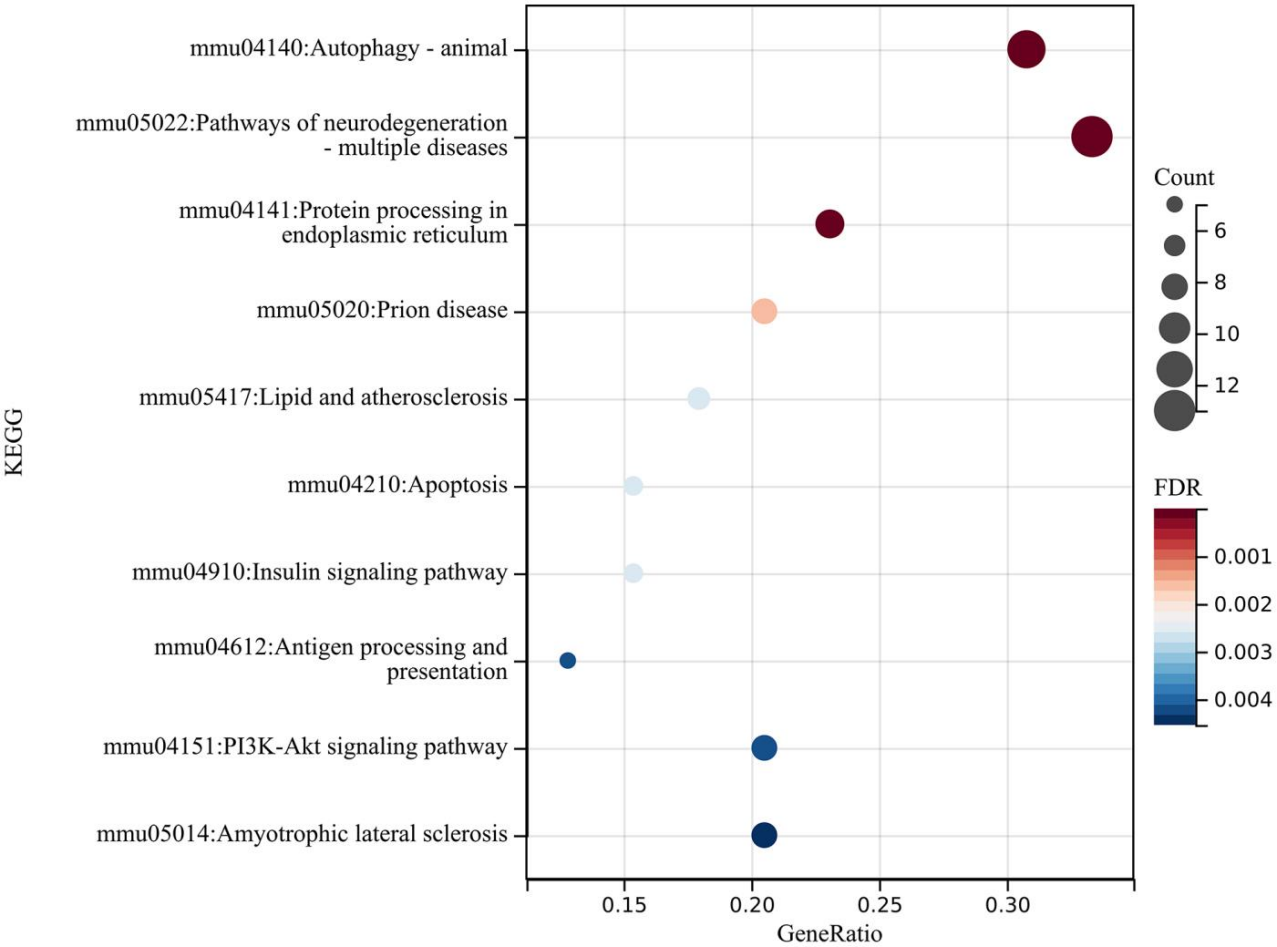
**The mRNA analysis.** Performed KEGG analysis on these differentially expressed genes to obtain relevant enrichment items.

**Figure 10:**
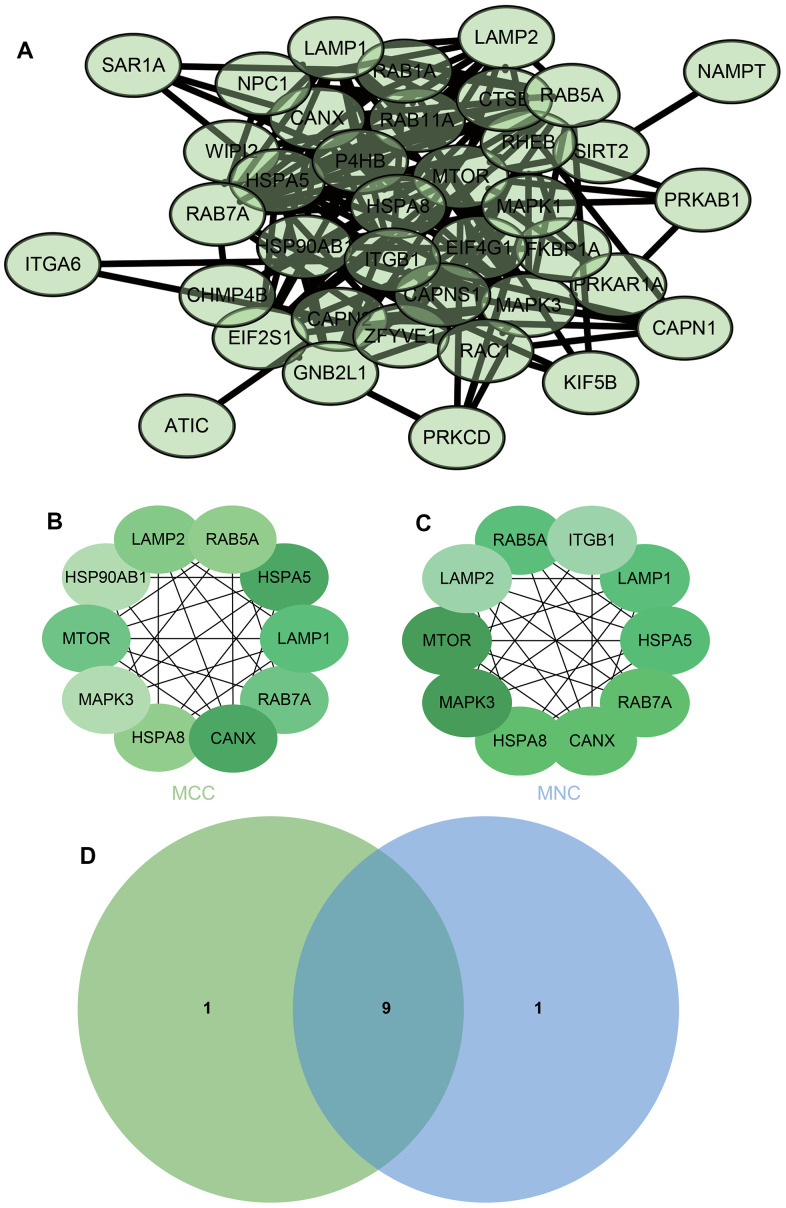
The mRNA analysis (**A**) PPI network (**B**, **C**) using two algorithms (MCC, MNC) to identify hub genes (**D**) Venn diagram to obtain a Union.

We then performed KEGG analysis on these differentially expressed genes to obtain relevant enrichment items KEGG analysis of DEGs was performed to obtain related enrichment entries (Figure 9), and entered these differentially expressed genes into the STRING website to create a PPI network (Figure 10A), using two algorithms (MCC, MNC) to identify hub genes (Figure 10B), followed by a Venn diagram to obtain a Union (Figure 10C), Core genes related to autophagy mRNAs were obtained (HSPA5, CANX, LAMP1, mTOR, RAB7A, LAMP2, RAB5A, HSPA8, MAPK3).

### CTD analysis

**Figure 11:**
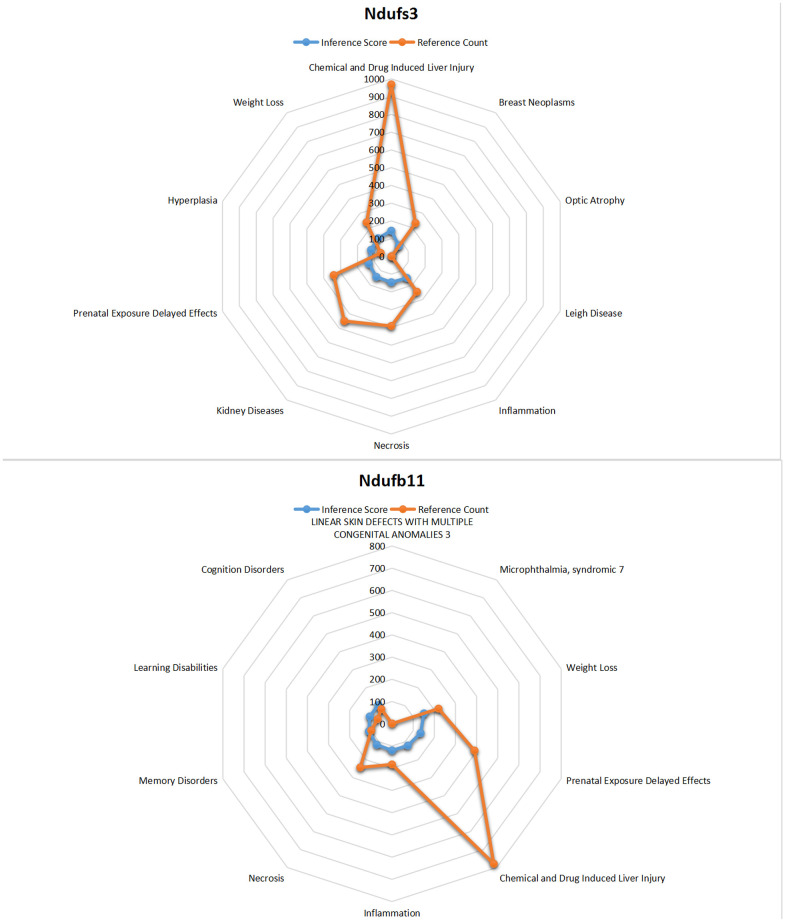
**CTD analysis.** The core genes NDUFB11 and NDUFS3 were found to be associated with necrosis, hyperplasia, inflammation, renal disease, weight loss, memory impairment, and cognitive impairment.

Core genes was entered into CTD to find diseases related to core genes. The core genes NDUFB11 and NDUFS3 were found to be associated with necrosis, hyperplasia, inflammation, renal disease, weight loss, memory impairment, and cognitive impairment (Figure 11).

### WB

The expression level of NDUFS3, E-cadherin, BCL2, P21 and SOX9 in atherosclerosis and chronic stress were lower than that in control group. They were higher in AS+CS/NDUFS3-OE group than in AS+CS group. The expression was lower in the AS+CS/NDUFS3-OE group than in the AS+CS group.

**Figure 12:**
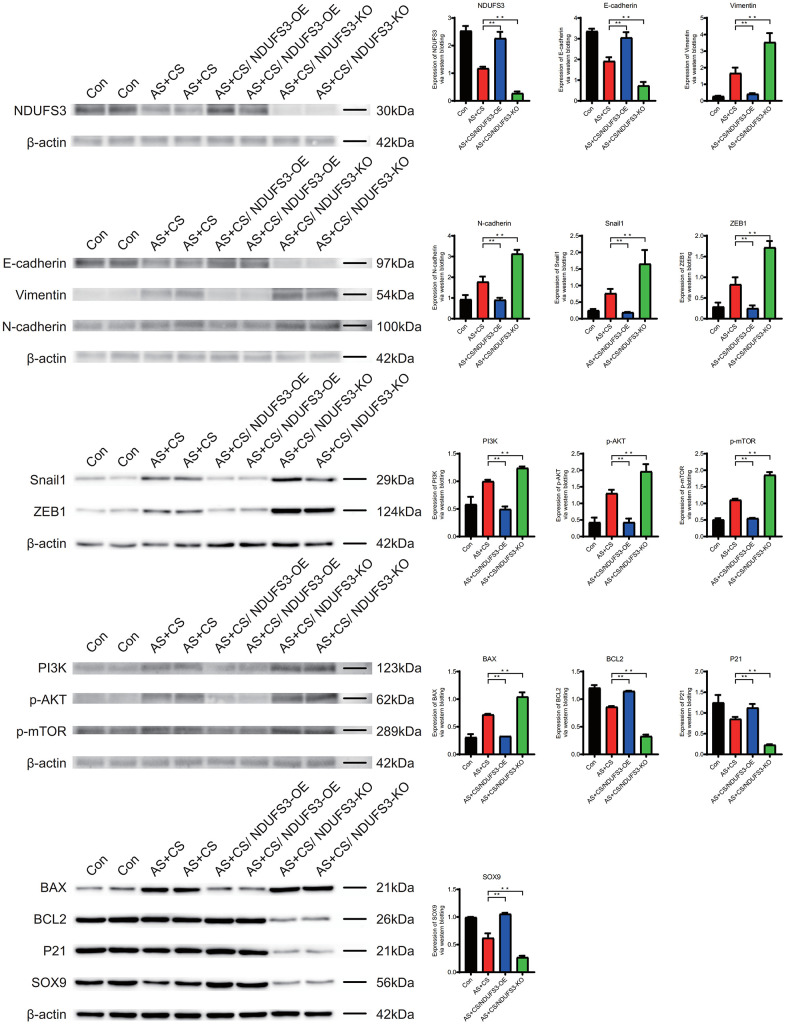
The expression level of NDUFS3 in the atherosclerosis and chronic stress groups was lower than that in the control group.

The expression level of Vimentin, N-cadherin, Snail1, ZEB1, PI3K, p-AKT, p-mTOR and BAX in atherosclerosis and chronic stress were higher than that in control group. They were lower in AS+CS/NDUFS3-OE group than in AS+CS group. The expression was higher in the AS+CS/NDUFS3-OE group than in the AS+CS group (Figure 12).

### The miRNAs prediction and functional annotation associated with core genes

The hub genes were entered into TargetScan to search for relevant miRNA (Table 1). The related miRNAs of NDUFS3 gene are mmu-mir-125b-5p, mmu-mir-6394, mmu-mir-6367.

**Table 1:** A summary of miRNAs that regulate hub genes.

	**Gene**	**MIRNA**		
1	**Ndufs3**	mmu-miR-125b-5p	mmu-miR-6394	mmu-miR-6367
2	**Ndufb11**	none		

## DISCUSSION

Atherosclerosis is a chronic and progressive disease in which plaques that form become occluded within the arterial wall reduce blood flow, increase blood pressure, and trigger complications such as vascular rupture, thrombosis, and embolization, leading to diseases such as glomerulosclerosis, renal insufficiency, and renal failure [12]. Chronic stress may lead to mental health problems such as mood disorders, depression, anxiety and insomnia, resulting in decreased function of the immune system and increased risk of infections, inflammatory and autoimmune diseases, metabolic disorders, peptic ulcer inflammatory bowel disease and other digestive diseases, infertility, sexual dysfunction and other reproductive diseases [13, 14]. Several molecular mechanisms are involved in the development and progression of atherosclerosis, including oxidative stress, inflammatory response, platelet activation, abnormal cholesterol metabolism, and apoptosis [15–17]. The molecular mechanisms of chronic stress involve disturbances in several biological systems [18–20]. The main results of this study are that NDUFB11 and NDUFS3 are underexpressed in atherosclerosis and chronic stress, and the lower the NDUFB11 and NDUFS3, the worse the prognosis.

NDUFB11 is one of the subunits of complex I of mitochondrial respiratory chain and is a key factor in the electron transfer process [21]. Mitochondrial respiratory chain complex I, contains multiple subunits and cofactors and is required for maintaining the energy metabolism of mitochondria. NDUFB11 is mainly involved in complex I assembly and function stability, but also in regulation of respiratory function, apoptosis, and oxidative stress response in mitochondria [22]. Recent studies have shown that mutations in NDUFB11 can be associated with the development of many diseases. For example, NDUFB11 deficiency was found to be a rare mitochondrial disorder known as NDUFB11 deficient mitochondriopathy. Patients with this disease usually present with a variety of symptoms, including muscle weakness, neurological disorders, and metabolic disorders, among others. NDUFB11 expression levels may also be involved in the development of other diseases, such as tumors, neurodegenerative and cardiovascular diseases, among others [23]. NDUFB11 mutations all have an established role in the mitochondrial respiratory chain [24]. The NDUFB11 gene is associated with neurogenetic disorders [25].

NDUFS3 is a subunit of mitochondrial respiratory chain complex I [26, 27]. Mitochondrial respiratory chain complex I, a complex in the mitochondria, is involved in the process of oxidative phosphorylation in cells. NDUFS3 is one of the key subunits of this complex responsible for important steps in the electron transport process, and its absence or aberrant function is associated with disorders associated with mitochondrial respiratory chain disorders, such as muscular, neurodegenerative, and metabolic diseases, among others. The study of NDUFS3 may provide insight into mitochondrial function and oxidative phosphorylation processes and shed new light on the treatment of related diseases [27, 28]. Therefore, we speculate that NDUFB11 and NDUFS3 may play important roles in intracellular energy metabolism in atherosclerosis and chronic stress.

Although this paper has carried out rigorous bioinformatics analysis, there are still some shortcomings. Animal experiments with overexpression or knockdown of the gene were not performed in this study to further verify the function.

In summary, NDUFB11 and NDUFS3 are underexpressed in atherosclerosis and chronic stress, and the lower NDUFB11 and NDUFS3, the worse the prognosis. NDUFB11 and NDUFS3 may serve as molecular targets for the precision treatment of atherosclerosis and chronic stress, thus providing a foundation of direction for mechanistic studies of atherosclerosis and chronic stress.

## MATERIALS AND METHODS

### Atherosclerosis accompanied by chronic stress dataset

In this study, the atherosclerosis with chronic stress group data file including 3 atherosclerosis with chronic stress samples and 3 normal samples.

### Screening of differentially expressed genes (DEGs)

Probe aggregation and background correction of merge matrix of atherosclerotic concomitant chronic stress using R package “limma”. P value were adjusted using Benjamini-Hochberg method. The fold change (FC) is calculated using false discovery rate (FDR). The cutoff value of DEG is p less than 0.05 and FC is greater than 1.5. And make a visual representation of the volcano.

### Weighted gene co expression network analysis (WGCNA)

First of all, use de-batch and post-merge matrix of atherosclerosis accompanying chronic stress to calculate median absolute deviation (MAD) of each gene. Outlier genes and samples were removed by good sample gene method of WGCNA in R package, and scale-free co-expression network was constructed. We calculated characteristic gene differences of modules, and selected tangent line for module tree view, incorporated part of modules.

### Construction and analysis of protein-protein interaction (PPI) networks

Search Tool for the Retrieval of Interacting Genes (STRING) is a search system for known and predicted PPI. STRING database also contains the predicted results using bioinformatics methods. The differential genes were input into STRING to construct PPI network and predict core genes. PPI network was visualized, core genes are predicted by Cytoscape software. First of all, we import PPI network into the Cytoscape, and then find module with the best correlation through MCODE, and MCC and MNC were used to calculate the best correlated genes. Finally, the list of core genes was obtained after visualization.

### Functional enrichment analysis

Gene Ontology (GO) analysis is a computational method to evaluate gene functions and biological pathways, and it is a key step to endow sequence information with practical biological significance. Kyoto Encyclopedia of Gene and Genome (KEGG) is an online database dedicated to collecting information on genomes, molecular interaction networks, enzyme catalytic pathways, and biochemical products. The genomic information and gene function were linked, and gene function was systematically analyzed. The list of differential genes screened by Wayne map was input into KEGG rest API obtained latest KEGG Pathway gene annotation. Gene set enrichment results were obtained using R package cluster Profiler.

Metascape (http://metascape.org/) can realize cognition of gene or protein function, and can be visually exported. We used Metascape database to analyze functional enrichment of the above differential gene list and derive it.

### Gene set enrichment analysis (GSEA)

The samples were divided into two groups according to the atherosclerosis accompanying chronic stress and normal groups, and the data were GSEA is based on level-specific gene probes that evaluate data from microarrays and is a way to uncover genomic expression data through fundamental knowledge. The samples were divided into atherosclerosis accompanying chronic stress and normal groups. 5 is minimum gene set and 5000 is maximum gene set, 1000 resampling times.

### Gene expression heatmap

We use R-packet heatmap to map expression of core genes found in PPI network, and to visualize difference of core gene expression between atherosclerosis accompanying chronic stress and normal samples.

### Immune infiltration analysis

The CIBERSORT (http://CIBERSORT.stanford.edu/) is a very common method for calculating immune cell infiltration. We applied the integrated bioinformatics method, used the CIBERSORT software package to analyze the de-batch merging matrix of atherosclerotic concomitant chronic stress, and immune cell abundance was estimated by deconvoluting the expression matrix of immune cell subtypes by linear support vector regression principle. At the same time, the samples with sufficient confidence were selected by using confidence P < 0.05as the truncation criterion.

### The messenger RNA (mRNA) analysis

We intersected with the screening derived differential genes by extracting autophagy related genes in the matrix of atherosclerosis with chronic stress for identification of differentially expressed genes related to autophagy.

### Comparative toxicogenomics database (CTD)

The CTD (https://ctdbase.org/) is a powerful public database that links toxicological information on chemicals, genes, phenotypes, diseases, and exposures to advance understanding of human health. CTD incorporates literature based, manually curated interactions to create a knowledge base that orchestrates chemical exposures and their biological effects across heterogeneous data across species. CTD identifies integrated chemical diseases, chemical genes, and gene-disease interactions that are used to predict disease-related gene/protein relationships as well as new associations and generate extended networks. We input core gene into CTD, find disease most related to core gene, and drew an expression difference radar plot for each gene with Excel.

### WB

Western blotting, also known as immunoblotting, is a method to detect the expression of a certain protein in complex samples according to the specific binding of antigens and antibodies, and can qualitatively and semi-quantitatively analyze proteins. Total protein was extracted and the protein content was determined. After SDS-PAGE electrophoresis and membrane transfer, the protein samples were blocked with 5% skim milk for 1 h at room temperature, shaken with Tris Buffered Saline Tween at high speed on a shaker, washed for 5 min, and repeated three times. The primary antibody was added and incubated overnight at 4° C, followed by TBST shaking 3 times (5 min each time) and TBST shaking 3 times (5 min each time). The results were analyzed after chemiluminescence development.

### miRNA

TargetScan (https://www.targetscan.org) can predict and analyze miRNA and target genes. Screening of miRNAs regulating central DEGs was performed using TargetScan in this study.

### Availability of data and materials

The datasets generated during and/or analyzed during the current study are available from the corresponding author on reasonable request.
